# Repurposing Sewage and Toilet Systems: Environmental, Public Health, and Person‐Centered Healthcare Applications

**DOI:** 10.1002/gch2.202300358

**Published:** 2024-05-11

**Authors:** Defne Yigci, Joseph Bonventre, Aydogan Ozcan, Savas Tasoglu

**Affiliations:** ^1^ School of Medicine Koç University Istanbul 34450 Türkiye; ^2^ Division of Renal Medicine Department of Medicine, Brigham and Women's Hospital Harvard Medical School Boston MA 02115 USA; ^3^ Electrical and Computer Engineering Department University of California Los Angeles CA 90095 USA; ^4^ Bioengineering Department University of California Los Angeles CA 90095 USA; ^5^ California NanoSystems Institute (CNSI) University of California Los Angeles CA 90095 USA; ^6^ Computer Science Department University of California Los Angeles CA 90095 USA; ^7^ Department of Surgery David Geffen School of Medicine University of California Los Angeles CA 90095 USA; ^8^ Department of Mechanical Engineering Koç University Sariyer Istanbul 34450 Türkiye; ^9^ Koç University Translational Medicine Research Center (KUTTAM) Koç University Istanbul 34450 Türkiye; ^10^ Boğaziçi Institute of Biomedical Engineering Boğaziçi University Istanbul 34684 Turkey; ^11^ Koç University Arçelik Research Center for Creative Industries (KUAR) Koç University Istanbul 34450 Turkey

**Keywords:** health monitoring, toilet‐based sensors, urine, wastewater management, water scarcity

## Abstract

Global terrestrial water supplies are rapidly depleting due to the consequences of climate change. Water scarcity results in an inevitable compromise of safe hygiene and sanitation practices, leading to the transmission of water‐borne infectious diseases, and the preventable deaths of over 800.000 people each year. Moreover, almost 500 million people lack access to toilets and sanitation systems. Ecosystems are estimated to be contaminated by 6.2 million tons of nitrogenous products from human wastewater management practices. It is therefore imperative to transform toilet and sewage systems to promote equitable access to water and sanitation, improve public health, conserve water, and protect ecosystems. Here, the integration of emerging technologies in toilet and sewage networks to repurpose toilet and wastewater systems is reviewed. Potential applications of these systems to develop sustainable solutions to environmental challenges, promote public health, and advance person‐centered healthcare are discussed.

## Graphical abstract

The integration of emerging technologies in toilet and sewage networks can repurpose toilet and wastewater systems and facilitate the development of sustainable solutions to global issues. Here, environmental, public‐health related, and person‐centered healthcare applications of repurposed toilet and sewage networks are reviewed. Potential challenges in implementation and future directions are discussed.

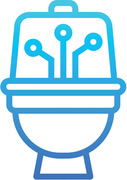

## 1 Introduction

**Figure 1:**
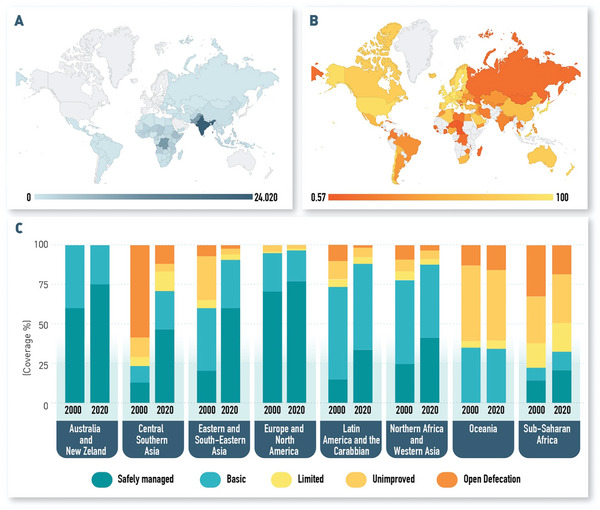
Overview of the global impact of the lack of access to adequate sanitation and safely managed toilet or sewage networks. A) Number of diarrhea‐related deaths of children under 5 years old (<5) attributed to inadequate water in 2019. Data acquired from World Health Organization (WHO) Global Health Observatory.^[^ ^4^ ^]^ B) Proportion of safely treated domestic wastewater (shown as percentage of wastewater flows that are safely treated). Data acquired from World Health Organization (WHO) Global Health Observatory.^[^ ^5^ ^]^ C) Proportion of population with access to sanitation facilities in Sustainable Development Goals (SDG) regions in 2000 and 2020. Data acquired and adapted from WHO/UNICEF Joint Monitoring Program (JMP).^[^ ^6^ ^]^

The burden of climate change, depletion of natural resources, and rapid growth of the global population have exacerbated pre‐existing health inequities. The lack of access to safe drinking water is estimated to cause the preventable death of almost 297.000 children (<5 years of age) and 829.000 people each year.^[^ ^1^ ^]^ Water scarcity results in a compromise of safe hygiene practices, leading to the contamination of water sources, and the transmission of diseases like cholera, infectious diarrhea, dysentery, and hepatitis A in water‐stressed areas where 2 billion people reside (**Figure** **1**A). In addition, millions of people are impacted by neglected tropical diseases and parasitic infections associated with fecal‐soil contamination.^[^ ^2^ ^]^ It is therefore imperative to not only ensure global access to safe water but also to implement water recycling, management, and conservation strategies to enable sustainable solutions for low‐resource settings. Building adequate sanitation systems and infrastructure is the first step in ensuring access to safe water.

Implementation of toilet and sewage networks is nothing new. In fact, the first recorded wastewater management applications date back to 4000 BC, where drainage systems were constructed for stormwater collection; vaulted sewers enabled the removal of household waste; urban drainage systems were developed as uncleanliness was associated with evil; and openings in the floor termed cesspools were used as toilets by the Babylonians.^[^ ^3^ ^]^ Later, plumping systems that employed hydraulics, channelized drains made of terracotta pipes, and wooden toilet stools were developed in or around palaces and courts by the Minoans between 3000–1000 BC. Similarly, copper piping systems and limestone toilets were constructed in pyramids by Ancient Egyptians in 2000–500 BC. It was not until the Hellenistic period (480–67 BC) that the correlation between water sanitation and public health was established and organized bath, toilet, sewage, and house drain systems emerged. By the 14^th^ century, in medieval Europe, rivers still served as open sewers and the lack of adequate infrastructure for wastewater management resulted in several enteric disease outbreaks and epidemics in Europe until the 19^th^ century.

Since then, technological advancements have largely transformed modern toilet and sewage systems in developed countries, enabling the safe treatment of wastewater (Figure 1B). However, in 2020, 494 million people still did not have access to toilets and practiced open defecation and 3.6 billion people were unable to practice safe sanitation at their homes (Figure 1C).^[^ ^2^ ^]^ Therefore, a dire need for interdisciplinary and innovative strategies to repurpose toilets and sewage networks exists. Strong strategies should entail several components and 1) prioritize cost‐effectiveness, sustainability, and equity; 2) maximize water conservation; 3) prevent water or soil contamination; 4) integrate smart technologies to facilitate higher functionality (i.e., processing and disease or hygiene tracking). Here, strategies used to repurpose toilet and sewage networks are reviewed. The integration of emerging technologies in such systems is covered. Current challenges in widespread implementation, potential applications to address environmental challenges, implications for public health, and future directions in person‐centered healthcare are discussed.

## 2 Sustainable Solutions to Environmental Challenges

The compounding effects of climate change are expected to decrease global terrestrial water storage significantly by the end of the 21^st^ century, implying that the severity and frequency of droughts particularly in the Southern hemisphere will continue to increase.^[^ ^7^ ^]^ This not only poses an imminent risk on water and food security, but also threatens terrestrial aquatic habitats and river biodiversity.^[^ ^8^ ^]^ Less wealthy nations remain particularly vulnerable to the global consequences of depleting water resources.^[^ ^9^ ^]^ Balancing ecological sustainability, water security, and agricultural demands of the century requires water management policies and strategies guided by effective water optimization frameworks. Such strategies include prevention of ecosystem contamination, wastewater repurposing, and water recycling.

### 2.1 Preventing Ecosystem Contamination and Repurposing Urine

**Figure 2:**
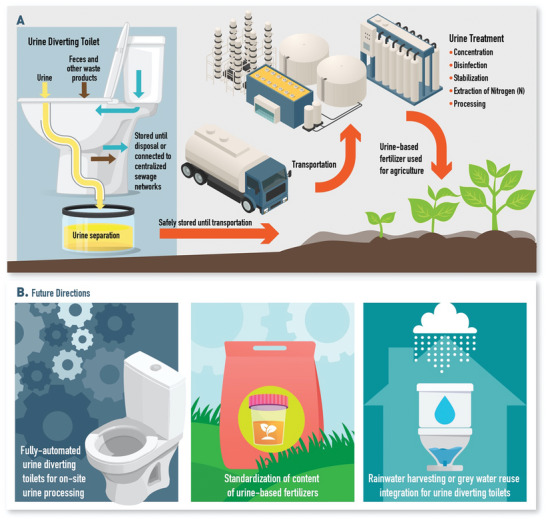
Urine separation and repurposing urine. A) Urine based fertilizer production workflow. Urine diverting toilets can be employed for urine separation from feces and other waste products. Isolated urine can be safely stored and transported to treatment facilities where fertilizers are generated. B) Future directions for urine‐based fertilizer production. Fully automated urine diverting toilets can reduce costs and transportation requirements by enabling on‐site processing. Fertilizer content standardization can increase crop yield. Rainwater harvesting and greywater recycling can be integrated in urine diverting toilets to increase water conservation. Some elements in this figure have been created using resources from freepik.com.

6.2 million tons of nitrogenous products (most of which originates from urine) are estimated to contaminate coastal ecosystems every year due to human wastewater discharge practices.^[^ ^10^ ^]^ Conventional toilets rely on flushing systems: urine, feces, water, and paper are all collected at centralized sewers wherever infrastructure permits.^[^ ^10^, ^11^ ^]^ As a result, ecological contamination remains inevitable and valuable opportunities to repurpose wastewater are overlooked. Urine separation can not only reduce ecological contamination, but also allow urine to be collected, treated, and reused. The composition of urine is rich in nitrogen (N), phosphorous (P), and potassium (K), making it an ideal candidate for use as an agricultural fertilizer.^[^ ^12^ ^]^ In fact, the potential use of urine as an agricultural fertilizer has been studied extensively since the 1990s and depends on the isolation and treatment of urine. As it is challenging to isolate urine from wastewater once it has been contaminated with feces, urine isolation strategies have focused on reimagined toilet designs (**Table** **1**). Initial designs of urine diverting dry toilets (UDDTs) successfully separated urine from feces using vaults albeit the unorthodox design.^[^ ^11^ ^]^ Installed in 2002, by the eThekwini Municipality in South Africa in response to a cholera outbreak, these UDDTs tremendously increased access to safe sanitation in the area. However, the flush‐free design failed to eliminate odor and inexpensive construction resulted in malfunctioning pedestals and low user satisfaction.^[^ ^13^ ^]^ Moreover, UDDTs have been considered an unjust solution designed solely for economically disadvantaged communities, deepening perceived disparities in South Africa. As such, for community‐based applications, solutions must adopt a user‐centered and community‐up approach instead of a top‐down one. In addition, it has been demonstrated that self‐flushing toilets offer better on‐site sanitation for prolonged use compared to urine diverting counterparts as self‐flushing toilets provide improved bacterial disinfection, reduce odors, can degrade organics.^[^ ^14^ ^]^ In 2017, the NoMix toilet was able to address some of these challenges by exploiting the hydraulic teapot phenomenon to alter toilet curvature and separate urine into a “urine trap” without valves, vaults, sensors, or compromising on the comfort of flushing systems (**Figure** **2**).^[^ ^15^ ^]^ The urine trap had an intuitive and conventional design, isolated urine, and reduced flush water contamination to less than 2.5%. However, it required water for flushing. Additionally, when no‐mix toilets leveraging the teapot phenomenon were installed in shared bathrooms, it was demonstrated that urine and nitrogen separation was influenced by hygiene practices.^[^ ^16^ ^]^

**Table 1:** Smart toilet designs for environmental and neighborhood‐based applications.

Design	Goal	Advantages	Limitations	Refs.
NoMix Toilet using the hydraulic teapot phenomenon	Urine separation	– Less than 2.5% flush water enters urine container – No user behavior change required – High urine separation	– Water conservation is low	[15]
Container‐based UDDT with integrated ash treatment	Urine based dry fertilizer production	– Offensive odor not detected – All‐in‐one separation, storage, treatment and reduction of urine – Dry powdered final product N, P, and K content equivalent to commercial fertilizer	– High temperature (65 °C) required for faster and more robust treatment	[20]
Alkaline dehydration based UDT	Urine based dry fertilizer production	– Scaling up is possible – Dry product recovery	– High salt concentration of final product – High energy demand	[21]
IoT enabled public toilet	Disease detection	– Alerts next user if toilet is unsafe/ unhygienic – Automated testing	– No integrated cleaning system – Clear informed consent process should be added before commercial use	[23]
Emergency sanitation operation system (so) with sensors and information communication technologies (ICT)	Emergency response for sanitation needs	– 97% water conservation – Safe excreta handling – Self‐cleaning (UV) – Urine and feces separation – Graywater collection	– Malfunctioning odor trap following a few days of use – Max. 30 persons/day for long‐term and max. 200 persons/day for short‐term use	[24]

While urine separation can prevent ecosystem contamination to a great extent, it is only the first step to repurposing wastewater (Figure 2A). The use of urine without further processing remains notoriously difficult as the nitrogen concentration in urine is low (for fertilizers) and when urea reacts with water, a highly volatile pollutant, ammonia, is formed.^[^ ^17^ ^]^ Therefore, following separation, urine must be stabilized and concentrated to extract nutrients for agricultural use. Several strategies have been employed to achieve this. Magnesium or calcium dosing achieved high phosphorous content; acidification and alkalinization of urine inhibited urease, and thereby the conversion of urea to ammonia, resulting in a high nitrogen concentration; ammonia stripping, a process in which ammonia is first evaporated and then recollected using pH and temperature manipulations, was employed; zeolite or activated carbon adsorption collected ammonia on modified surfaces; and water extraction was done to concentrate urine. Processing eliminated most pathogenic bacteria within days and most viruses within weeks, yet, some pathogenic microorganisms (*Ascaris* or Clostridia) could persist for months in stored urine and pharmaceutical contamination remained a challenge.^[^ ^18^ ^]^ Therefore, it has been recommended by the WHO that urine must be stored for >6 months (T > 20 °C) prior to application.^[^ ^19^ ^]^ In addition, any urine treatment requiring heat‐based applications and/or addition of expensive chemicals, increased the cost and energy consumption, threatening the commercial viability of these strategies. Despite these challenges, integration of urine treatment methods into UDDTs has proved promising. Wood ash at 35 and 65 °C was used to alkalinize urine and achieved 95% mass reduction of urine, generating an 8% N, 2.5% P, and 10.9% K by weight dry product, which is equivalent to the composition of commercial fertilizers.^[^ ^20^ ^]^ This construct also eliminated the need for liquid dispersal and transportation challenges for UDDTs, thereby offering an ease of application particularly for low resource areas. Alternatively, a pilot study was conducted at a military training camp in Finland to assess the viability of alkaline dehydration of source‐separated urine and end products with 1.4% N, 0.9% P, and 8.3% K content was produced.^[^ ^21^ ^]^ Although N recovery was expected to increase if the system worked at full design capacity and nutrient composition of the final product was similar to that of blended fertilizers, high salt content of the final product raised concerns for soil salinization. Additionally, high energy consumption was reported. Alternatively, artificial fresh urine was extracted using an Mg–air fuel cell integrated into a water‐free urinal to generate artificial phosphate ore to be used as a fertilizer.^[^ ^22^ ^]^ It was estimated that $2.58 net profit could be achieved for 1 m^3^ of undiluted urine, proposing an economically feasible application.

### 2.2 Water Conservation

In addition to preventing ecological contamination and repurposing urine content to make fertilizers, toilet, and sewage systems can be redesigned to optimize water conservation. Besides UDDTs, low‐flush toilets (i.e., vacuum toilets), greywater (wastewater acquired from domestic applications except toilet flushing) reuse (GWR), rainwater harvesting (RWH) have been pursued to optimize water usage.^[^ ^25^ ^]^ Low‐flush toilets can conserve water and GWR could be salvaged for irrigation and toilet flushing purposes to functionalize water that would otherwise be wasted. While GWR could significantly reduce urban freshwater demand, treatment and continuous monitoring are necessary to prevent pathogen spread, soil alkalinization, and groundwater contamination.^[^ ^26^ ^]^ Another hurdle relating to the widespread implementation of low‐flush toilets and GWR is the risk for sedimentation and blocking in sewage systems.^[^ ^27^ ^]^ Decentralized RWH could mitigate this issue and maintain high water conservation through two systems: a rooftop system to collect rainwater and a run‐off system to collect contaminated rain and storm water.^[^ ^28^ ^]^ Harvested rainwater could be used for flushing or recycling. However, in the United States, decentralized RWH offered little economic appeal over centralized systems.^[^ ^29^ ^]^ To overcome this issue, a GWR and RWH integrated system was built in Durban, Kwa‐Zulu Natal province of South Africa.^[^ ^30^ ^]^ The integrated pump and tank system offered 50% annual cost savings and a 5‐year payback period, sustained residential water demand, and minimized dependence on electricity supply. It is important to note that water availability, water prices, rainfall, and access to water differ significantly among countries. As a result of these discrepancies, even if similar water collection systems are installed, household savings and efficiency rates can vary among different geographical locations. Additionally, central installation of such collection systems relies heavily on preexisting infrastructure. Several water‐stressed countries with adequate infrastructure have maintained centralized water conservation by reusing wastewater for agriculture following extensive treatment and disinfection. In Israel and Spain 86% and 77% of the country's sewage is used for agricultural purposes, respectively.^[^ ^31^ ^]^

## 3 Public Health Promoting Neighborhood‐Based Applications

It is also vital to ensure widespread testing of water since the lack of access to safe water is a dire problem. Even in the past decade, several instances of water contamination have been recorded globally: Drinking water was contaminated with lead and possibly *Legionella* in Flint, Michigan (USA) in 2014, exposing thousands of residents to potentially severe long‐term health consequences^[^ ^32^ ^]^; wastewater treatment plants contaminated water sources (surface and sea water) in Africa and pharmaceuticals, endocrine‐disrupting chemicals, personal care products, pesticides, per‐ and polyfluoroalkyl compounds, and microplastics have been detected in water sources^[^ ^33^ ^]^; fecal contamination of water has been estimated to cause 485.000 deaths each year.^[^ ^1^ ^]^ Moreover, heavy metal pollution, polluted water runoff, and wastewater contamination pose an imminent threat to human health and can result in the spread of infectious diseases and expose individuals to carcinogens or other toxic substances.^[^ ^34^ ^]^ Thus, it is imperative to provide continuous and widespread water testing and treatment.

### 3.1 Water Testing

Magnetic nanomaterial‐based sensors, electrochemical biosensors, solid‐phase or liquid‐phase extraction, spectrophotometry, adsorbent‐based methods, gas or high‐performance liquid chromatography, mass spectrometry, chemical precipitation, and chlorination have been used to treat water sources to ensure safety.^[^ ^35^, ^36^ ^]^ However, methods involving chemical precipitation have generated hazardous waste and non‐automated strategies have been limited by high‐costs and trained personnel requirements. Some cost‐effective and environmental friendly technologies for water testing include solar disinfection, filtration, and treatment of harvested rainwater.^[^ ^37^ ^]^ As solar disinfection efficiency is influenced by many factors including container type, weather, and water quality, additives have been used in conjunction with solar disinfection to enhance its functionality. Similarly, while many filters have been constructed, disinfecting against *Legionella pneumophila* has proved challenging. As such, no silver bullet currently exists to deliver environmentally friendly, low‐cost, and highly efficient and effective water treatment. Therefore, accurate, rapid, and robust water testing is necessary. To address some of these concerns, transduction‐based techniques (i.e., colorimetric or photoluminescence sensors) have emerged for heavy metal testing.^[^ ^38^ ^]^ Similarly, paper‐based applications have offered inexpensive and environmentally friendly testing but have not yet gained widespread acceptance. Organic linkers, metal nanoparticles, carbon quantum dots, semiconductor quantum dots have been used for detection and color charts, various scanner, and smartphones have allowed simple readout visualization. Alternatively, naturally bioluminescent bacteria like *Vibrio fischeri* have been used to detect stressors that can induce DNA damage; albeit with less sensitivity than pollutant‐specific tests, steroid receptors (i.e., androgen receptor, estrogen receptor) have been used to detect endocrine agonist and antagonists in water samples; and multiple tests including genotoxicity, cytotoxicity, and fish embryo toxicity have been conducted simultaneously to assess water content in wastewater treatment plants.^[^ ^39^, ^40^ ^]^

### 3.2 Wastewater Epidemiology

Another application for water testing that has gained momentum over the past decades is wastewater‐based epidemiology (WBE), which can offer rapid screening for pollutants and pathogens, thereby enabling rapid and widespread disease tracking.^[^ ^41^ ^]^ In addition to detecting exposure to carcinogenic chemicals like pesticides,^[^ ^42^ ^]^ phosphorous flame retardants and plasticizers,^[^ ^43^ ^]^ bisphenols,^[^ ^44^ ^]^ and illicit drugs (i.e., cocaine or amphetamines),^[^ ^45^ ^]^ WBE has also been employed to track the spread of infectious diseases.^[^ ^46^, ^47^, ^48^, ^49^ ^]^ To determine the prevalence of rotavirus group A and human adenovirus, 36 sewage and 96 surface water samples were collected from a wastewater treatment plant and along a drainage canal.^[^ ^50^ ^]^ Although infectivity of particles was not assessed, viral nucleic acids were extracted and then quantified using quantitative polymerase chain reaction (qPCR). While qPCR offers high sensitivity, it requires extensive lab infrastructure and therefore remains inappropriate for under‐resourced areas. Similarly, to determine the prevalence of Hepatitis B (HBV) in 19 cities in China, the concentration of lamivudine (an anti‐HBV, anti‐human immunodeficiency virus (HIV) drug) was investigated.^[^ ^51^ ^]^ Samples were collected from 92 municipal treatment plants; an ultrahigh performance liquid chromatography and a spectrometer were used following extraction. While results were consistent with HBV prevalence estimations, HIV patients using lamivudine were also included in these calculations, suggesting accuracy was lower than expected. Besides these applications, following the determination of viral shedding patterns of Severe Acute Respiratory Syndrome Coronavirus 2 (SARS‐CoV‐2), WBE has appeared as an inexpensive and rapid tool for robust SARS‐CoV‐2 surveillance.^[^ ^52^ ^]^ Moreover, infectious disease surveillance can alert public health authorities for potential outbreaks and help take necessary measures. Although unlikely to replace individual testing, WBE can reduce costs of testing for thousands of people and allow neighborhood‐based infectious disease tracking. However, wastewater infrastructure remains lacking in 60 countries and 5 billion people are not served by centralized sewing systems, significantly restraining efforts to use WBE for global disease surveillance.^[^ ^53^ ^]^

### 3.3 Toilet‐Based Applications

WBE has not yet been fully automatized and therefore applying WBE strategies for toilets in areas where centralized sewage systems are unavailable, remains expensive and time consuming. One way to mitigate this issue would rely on integrating infectious disease testing for UDDTs (Table 1). As feces and urine would already be separated, extraction procedures would be reduced. Alternatively, the development of fully automated smart toilets that can offer passive surveillance has been suggested.^[^ ^54^ ^]^ Moreover, automated nucleic acid amplification technique‐based detection of SARS‐CoV‐2 would follow fecal RNA isolation. These toilets would be installed at communal places and would require users to complete a quick response consent code prior to testing. However, this application could raise public concerns regarding data privacy. While the widespread use of fully automated toilet‐based pathogen detection could appear challenging for the time being, a public toilet utilizing Internet of things (IoT) was developed to detect bacteria and viruses in urine and alert users.^[^ ^23^ ^]^ For another toilet design prioritizing water conservation, a solar powered self‐cleaning automatic flushing system was developed.^[^ ^55^ ^]^ A bi‐slant conveyor belt was used to separate feces and urine to avoid blackwater contamination of the environment. An IoT based tracking system was also integrated in the design to detect sewage system blockages and contamination in order to solve a long‐lasting problem in India. Other toilets prioritizing fully automated sanitation or water conservation have also been developed. For example, an emergency sanitation operation system (eSOS) achieving 97% water savings has been constructed and was used in an emergency settlement in the Philippines.^[^ ^24^ ^]^ While the system collected data on usage patterns (i.e., time, frequency, user age group), the urine odor trap malfunctioned after a few days. Although these designs have appeared promising for a variety of settings (disaster zones, rural areas, urban crowded settings), cost effectiveness and development of regulations regarding data collection could delay the commercialization and widespread application of these technologies.

## 4 Person‐Centered Medicine

Apart from community‐based applications, toilet systems can also be transformed to enable person‐centered medicine applications. 41 million people die each year due to non‐communicable diseases (NCDs), including cardiovascular disease, diabetes, cancer, kidney disease, and chronic respiratory disease.^[^ ^56^ ^]^ Patients’ health in many chronic NCDs can deteriorate rapidly, especially if continuous and accurate health monitoring is not achieved. A significant proportion of healthcare currently relies on reactive intervention instead of prevention. As a result, the costs of healthcare are rapidly increasing, with $3.8 trillion and £225.2 billion spent in 2019 in the US and UK respectively.^[^ ^57^ ^]^ Healthcare costs have almost tripled in the UK over the past two decades (£78.9 billion had been spent 2000). A similar trend is observed in the US where healthcare expenditure is expected to reach $6.2 trillion by 2028. Furthermore, the need for better systems to offer proactive, prevention‐based, person‐centered care and better continuous health monitoring is evident. To achieve this, accessible health monitoring devices that can accurately collect physiological data and seamlessly integrate into daily routines can be utilized. An ideal health monitor 1) is noninvasive, 2) detects wide range of biomarkers with high accuracy, 3) is inexpensive, 4) offers simple result interpretation for non‐medical personnel, and (5) is fully automated.

### 4.1 Screening, Diagnosis, and Longitudinal Health Monitoring

**Figure 3:**
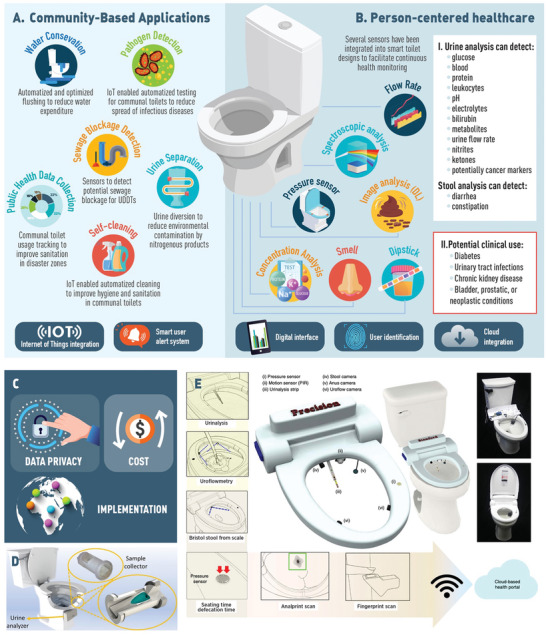
Reimagined smart toilets for community based and person‐centered healthcare applications. A) Proof‐of‐concept smart toilets designs for community‐based applications focusing on reducing water expenditure, improving hygiene, preventing the spread of infectious diseases, and rapidly detecting public health emergencies (i.e., wastewater overflow due to sewage blockage caused by UDDTs). B) Various sensors that have been integrated into smart toilet designs to enable continuous non‐invasive at‐home health monitoring. I) Urine content measured using these sensors and II) differential diagnoses. C) Considerations to enable widespread utilization of smart toilets for community‐based and person‐centered applications. Said applications are concerned with sensitive private information and regulations are necessary to protect users’ information. Cost efficiency is imperative to allow widespread manufacturing and usage. Widespread implementation would have substantial benefits for communities if these technologies were tailored to the specific needs of communities. D) Smart toilet design measuring protein concentration adapted from Ref.[58] under Creative Commons Attribution 4.0 International License. E) Mountable toilet system for health monitoring adapted from ref.[59] Some elements in this figure have been created using resources from freepik.com.

Urine contains approximately 4500 metabolites and urine analysis can provide information about 600 physiological or pathological conditions including diabetes, cancer, urinary tract infections, pregnancy, ovulation as well as drug metabolites.^[^ ^60^ ^]^ Smart toilets can offer the convenience of in‐home non‐invasive continuous health monitoring through fully automated urine analysis. Seamlessly integrated into the daily routine, urine analyzing smart toilets can enable continuous health monitoring for NCD patients, eliminate recall bias, and serve as a screening tool for healthy or at‐risk individuals. Moreover, in addition to the detection of hematuria, proteinuria, glucosuria, bilirubin content, pH, creatinine (**Figure** **3**), urine tests can identify markers associated with chronic kidney disorders, neurodegenerative disorders, renal cell carcinoma, bladder, prostate, nasopharyngeal, lung, hepatocellular, gastric, and colorectal cancer as well as microRNAs associated with breast, ovarian, and pancreatic cancer.^[^ ^61^ ^]^ Moreover, health monitoring and screening can not only decrease healthcare expenditure, but also significantly improve health outcomes.^[^ ^57^ ^]^ Early diagnosis of chronic kidney disease can increase the quality of life and significantly slow down the progression of disease, preventing end‐stage kidney injury.^[^ ^62^ ^]^ Similarly, a delay of just 3 months in the diagnosis of breast cancer can result in more advanced stage cancer and poor prognosis.^[^ ^63^ ^]^

Urine analyzing toilets have been developed using several strategies (**Table** **2**). A 3D printed sample collector was constructed to measure protein content in urine.^[^ ^58^ ^]^ Similarly, a flexible soft silicon box equipped with electrodes was developed to detect urine glucose, pH, potassium ion (K^+^), and sodium ion (Na^+^) concentration.^[^ ^64^ ^]^ Thin film cells leveraging midinfrared spectroscopy were designed to detect glucose concentration in urine.^[^ ^65^ ^]^ An electronic nose composed of eight metal oxide gas sensors was developed and differentiated between healthy, diabetic, and alcohol containing urine.^[^ ^66^ ^]^ Alternatively, a retractable dipstick was used to carry out at‐home urine analysis.^[^ ^67^ ^]^ Likewise, a self‐contained smart toilet employed deep learning to classify stool consistency according to the Bristol scale, traced urine color, and calculated urine volume and flow rate.^[^ ^59^ ^]^ Fingerprint recognition was introduced, and an encrypted cloud server was used to store data. While these advancements are promising and can offer ease of health monitoring, data privacy is an important concern. Although smart toilets could prove cost‐effective on the long run by enabling early diagnosis and encourage timely medical advice seeking behavior, minimizing the costs of construction is essential to allow equity in access to these tools. Another important consideration is optimization of tests to avoid false‐positive and false‐negative results and to avoid over‐ or under‐testing.

**Table 2:** Smart‐toilet applications for continuous health monitoring.

Design	Sample analysis	Metabolite tested	Accuracy	Potential application	Refs.
3D printed urine collector with hydrophobic surface coating and integrated automatized protein concentration analysis	Plate reader and commercial dipsticks	Protein (BSA, albumin from bovine skin)	0.87 g L^−1^ (for 1 g L^−1^ sample) and 0.09 g L^−1^ (for 0.01 g L^−1^ sample)	Long term cyclic monitoring (kidney disease)	[58]
Soft silicon S‐tube with flexible electrodes and Bluetooth circuit board	Ag/AgCl modified electrodes	Glucose K^+^ Na^+^ pH	0‐1.8 × 10^−3^ m range 1–64 × 10^−3^ m range 8–256 × 10^−3^ m range pH: 4–8 range	Daily urine analysis (diabetes, hypertension, renal function, metabolic syndrome)	[64]
Thin film ultrasonic liquid cell and container with BaF_2_ optical windows transmitting midinfrared spectroscopy	Optical coherence microscopy (OTC)	Glucose	Positive for samples 50, 100, 200 mg dL^−1^	Potential detection of glucose content— stability and repeatability of liquid cell is required for future applications	[65]
Metal oxide gas sensors for electronic nose with wireless controlling system	Principal component analysis (PCA)	H_2_ NH_3_ Organic solvent vapors Air contaminants LPG, VOG H_2_S Solvent vapors and odorous gases	95% variance between healthy, diabetic, and alcohol containing urine	Monitorization of urine alcohol or glucose content	[66]
Urine and feces separating and storing toilet with infrared sensors as well as an RGB sensor for dipstick readout utilizing IoT	Urine dipstick and spectrophotometric analysis	Leukocytes Nitrite Urobilinogen Bilirubin pH Blood Protein Ketone Glucose Specific gravity	N/A	Proposed as a mini‐medical check‐up tool	[67]
Pressure and motion sensor integrated toilet using colorimetric analysis for urine analysis and deep learning (DL) for excreta analysis, with fingerprint and analprint user identification, connected to an encrypted cloud server	Urine dipstick and video capture for color analysis at cloud serves Uroflowmetry Stool analysis with DL	Leukocytes Nitrite Urobilinogen Bilirubin pH Blood Protein Ketone Glucose Specific gravity Flow rate Stool consistency	Pearson's *r* = 0.92	Potential daily‐clinic: Standard urine content analysis Prostate and bladder functions	[59]

## 5 Future Directions

Reimagining toilet and sewage systems to address global challenges of water conservation, lack of access to safe sanitation, disease tracking, and person‐centered healthcare remains promising. Future directions include the global implementation of urine reproposing strategies and disease tracking applications as well as the development of equitable person‐centered healthcare applications. For example, the repurposing of urine can not only conserve water on a household‐level but can also have significant environmental impacts if implemented at large scale. To enable the widespread implementation of urine repurposing strategies, it is essential to assess the benefits and drawbacks of nutrient recovery from household wastewater. To this end, it has been demonstrated that nitrogen emissions to wastewater can be reduced significantly at Malmö city if 60% urine concentration is achieved.^[^ ^68^ ^]^ Similarly, if 15–30% of urine in Malmö city is recycled and repurposed, half the fertilizer demand in the city could be met. It has also been revealed that urine‐based fertilizers can reduce greenhouse gas emissions and water consumption but could result in higher acidification potential and particulate matter emissions.^[^ ^69^ ^]^ As such, certain obstacles must be overcome before the widespread implementation of these systems can be fully realized. It is essential to ensure standardization and optimization for both household‐level strategies and large‐scale interventions. In addition, particularly for disease tracking applications, applications should develop transparent data privacy, data security, and informed consent processes to safeguard patient or user rights.

### 5.1 Standardization and Optimization

Standardization and optimization are essential to the sustainability and success of urine diversion applications and smart toilet designs. Moreover, particularly for areas with inadequate or decentralized sewage infrastructure, a lack of standardization will lead to dismal consequences. Byproducts can further environmental damage and inadvertently impede efforts to improve sanitation.^[^ ^70^ ^]^ For applications recycling urine into fertilizers, the final product would have to meet standards of safety and consistently improve crop yield (Figure 2B). Similarly, standardization of smart toilets for at‐home non‐invasive health monitoring is crucial to avoid inaccurate results. Machine learning integration can help improve the accuracy when interpreting the data collected.^[^ ^59^ ^]^ In addition to standardization, novel toilet or sewage constructs must be optimized to minimize cost and byproducts, reduce energy consumption, and maximize efficiency. Furthermore, high costs restrain efforts in providing equitable access and mass production. High energy consumption can not only drive the cost up but also create pollution. For instance, for urine‐based fertilizer application, urine treatment could be completed on/ near site of collection to avoid transportation.^[^ ^71^ ^]^ Alternatively, membrane distillation can be used in conjunction with electrodialysis to generate electricity which can later be used to sustain energy requirements of on‐site urine treatments.^[^ ^72^ ^]^

### 5.2 Data Privacy and Informed Consent

Continuous health monitoring through smart toilets and neighborhood‐based disease tracking (i.e., disease tracking toilets in shared bathrooms) can offer significant benefits for patients and communities, yet acquiring informed consent for data collection should be on the forefront of innovation and data privacy must be maintained. Moreover, although many people readily give electronic consent, most have little understanding of the conditions they agree to.^[^ ^73^ ^]^ As such, not only is it crucial to provide transparency regarding data collection intent, but it is also imperative to establish strong mechanisms of regulation to protect user's rights. While complex cloud computing platforms, data encryption, or blockchain‐based strategies can mitigate the issue of data privacy to some extent, these technologies can remain expensive, complex, and challenging to regulate.^[^ ^57^ ^]^ Cloud computing data leaks remain a concern – considering that health information is particularly sensitive and can be used against patients by insurance companies; data encryption can complicate user experience; and permanent data retention on blockchain can threaten user confidence in these systems.

## 6 Conclusion

Rapidly depleting clean water resources, inequities in access to sanitation, the rapidly increasing population, and climate change pose an imminent threat on global public health and equity, warranting sustainable, innovative, and interdisciplinary solutions. Although challenges remain, reimagined toilets and sewage systems designed to facilitate environmental transformations, inform community‐based interventions, or allow continuous health monitoring for person‐centered care can help address several challenges of the 21^st^ century.

## Conflict of Interest

The authors declare no conflict of interest.
